# Meta-analysis of the utility of culture, biopsy, and direct KOH examination for the diagnosis of onychomycosis

**DOI:** 10.1186/s12879-017-2258-3

**Published:** 2017-02-22

**Authors:** Verónica Velasquez-Agudelo, Jaiberth Antonio Cardona-Arias

**Affiliations:** 1 Biological Research Corporation (Corporación para Investigaciones Biológicas - CIB), Medellín, Colombia; 2 Experimental Medical Mycology Research Group, CIB, Medellín, Colombia; 3 School of Microbiology, University of Antioquia (Universidad de Antioquia), Calle 67 Número 53 – 108, Bloque 5, oficina 103, Medellín, Colombia; 4 Faculty of Medicine, Cooperative University of Colombia (Universidad Cooperativa de Colombia), Medellín, Colombia

**Keywords:** Onychomycosis, Diagnosis, Validation studies, Test validity, Meta-analysis as topic

## Abstract

**Background:**

Onychomycosis is a highly prevalent disease worldwide. There is no standard test for its diagnosis, which remains costly, wasteful, and is sometimes delayed. The diagnostic tests for this disease are few and discordant. The objective was to evaluate the diagnostic validity, performance, and accuracy of culture, nail clipping with Periodic Acid-Schiff –PAS- staining (biopsy), and direct potassium hydroxide (KOH) examination for the study of onychomycosis.

**Methods:**

A systematic review was conducted via meta-analysis using 5 databases and 21 search strategies. An *ex ante* protocol was applied with inclusion and exclusion criteria. Quality was assessed with the Quality Assessment of Diagnostic Accuracy Studies (QUADAS) tool, and the sensitivity, specificity, predictive values, likelihood ratios, diagnostic odds ratios, receiver operating characteristic (ROC) curves, and proportion of correctly diagnosed patients were evaluated with the meta-analysis of studies of evaluations of diagnostic and screening tests (Meta-DiSc) and Epidat using a random effects model.

**Results:**

The efficiency or accuracy of the three tests is influenced by the methodological quality of the studies. These values are lower for KOH and culture and higher for biopsy in moderate quality studies.

**Conclusion:**

The diagnostic tests evaluated in this meta-analysis independently showed acceptable validity, performance, and efficiency, with nail clipping with PAS staining outperforming the other two tests.

## Background

Onychomycosis is a highly prevalent infection worldwide with a range between 2% and 30%, corresponding to 50% of nail diseases and 30% of superficial mycoses [1, 2]. Onychomycosis is a cosmopolitan disease, and its incidence increases according to age, climate, physical activity, occupation, and underlying diseases [3]. A higher prevalence has been reported in men, individuals over 60 years of age, patients with immunosuppressive diseases, such as human immunodeficiency virus (HIV) infection or immunological defects, diabetics, and patients with peripheral vascular disease [1]. Its incidence is also higher in humid and tropical climates, under poverty and overcrowding conditions, and in athletes or sportspersons in whom a higher incidence of tinea paedis has been reported [3, 4].

This disease occurs via fungal invasion of the nail. Over the development course of the infection, there is initial colonisation with subsequent invasion of the nail bed and plate that cause changes in the nail colour, texture, and shape. There are different clinical presentations, including distal subungual, proximal subungual, white superficial, and total onychodystrophy. The distal subungual form is the most common [3, 5].

In addition to the change in nail shape, onychomycosis is related to low self-esteem because those with the condition often experience shame at being associated with poor hygiene and as a source of transmission to other individuals in their surroundings. Onychomycosis is also related to economic and work-related problems, social rejection, and a decreased quality of life [5–8].

Causative agents are divided into the following three main groups: dermatophyte moulds, non-dermatophyte moulds, and yeasts. Dermatophyte moulds are moulds directly associated with the infection and clinical signs. The diagnostic value of the isolation of non-dermatophyte moulds or yeasts is controversial because it depends on the amount of inocula with positive growth in culture; moulds are microorganisms that can be present as skin colonisers (although they are transient colonisers in nails) and thus are often considered contaminants. In these cases, it is advised to repeat the procedure before considering them the infectious agents [6–8].

The tests used to establish the diagnosis of onychomycosis include direct potassium hydroxide (KOH) examination, culture, histopathology, confocal laser microscopy, phase-contrast microscopy, and state of the art techniques, such as Vitek, matrix-assisted laser desorption/ionization time-of-flight (MALDI-TOF) mass spectrometry, and polymerase chain reaction (PCR) [9]. Because some of these tests are expensive and require the use of specialised equipment and materials that are not routinely used, the most commonly used methods are direct KOH examination, culture, and to a lesser extent nail biopsy, which are simpler and less costly than confocal microscopy and MALDI-TOF.

Despite the technical advantages of the traditionally used tests for the detection of the causative agents, none can be considered as a standard test alone from the viewpoint of their diagnostic utility. Therefore, several criteria are typically used for diagnostic validity studies because their simultaneous use can increase the sensitivity and specificity [10]. However, there is currently no consensus on the most appropriate combination of tests because mixed results have been reported for performance and validity. In this regard, studies have shown a high variability of results in the application of individual tests or their combinations. Sensitivity values have been reported between 23% [11] and 84.6% [12] for culture, between 44% [13] and 100% [12] for KOH, and between 81% [14] and 91.6% [15] for biopsy. The sensitivity values reported for test combinations are 57% for biopsy and KOH [11] and 98.3% for biopsy and culture [15].

In addition to the high variability in individual results, another limitation of the available studies is the inclusion of small sample sizes. Studies have performed diagnostic evaluations with samples of 40 [12], 50 [13], 63 [11], or 96 [14] individuals who are usually not selected probabilistically. Similarly, most studies present an incomplete diagnostic assessment to the extent that only data on sensitivity, specificity and predictive values are reported and relevant parameters, such as likelihood ratios, the Youden index (J), and the receiver operating characteristic (ROC) curve, among others, are ignored. These limitations can be overcome by a meta-analysis of diagnostic tests. This type of study allows the calculation of these indices via comparison among studies, which shows the parameters related to the diagnostic utility in a greater number and different types of patients and identifies potential sources of heterogeneity of the results, among other advantages.

The objective of this study was to evaluate the diagnostic validity, performance, and accuracy of culture, nail clipping with PAS staining (biopsy), and direct KOH examination in the investigation of onychomycosis.

## Methods

### Type of study

Systematic review of the literature with a meta-analysis of diagnostic tests.

### Description of techniques

#### KOH examination

This method is a direct technique that can determine the presence of the microorganism by visual inspection. KOH degrades keratin, which allows better visualisation of fungal structures [1, 16].

#### Culture

Culture allows the isolation and detection of the aetiological agent and in most cases allows species differentiation. With this technique, it becomes essential to use a combination of different culture media to show fungal growth of the aetiological agent [1].

#### Nail clipping with PAS staining

Staining of a portion of the nail plate (nail biopsy) allows the identification of the ty13pe of fungal structure (hyphae or blastoconidia) and the degree of invasiveness according to the layer of the nail plate (inner, middle, or outer) in which the fungal structures are observed. The degree of invasiveness is directly related to the nail portion where the fungal structures are observed and is greater when the innermost layer is involved [1].

### Protocol for study search and selection according to the PRISMA (Preferred Reporting Items for Systematic Reviews and Meta-Analyses) criteria

#### Identification

A search for journal articles regarding sensitivity was performed. First, the terms were searched in DeCS and MeSH to identify all synonyms. This activity was complemented by Perl harvesting using the following selected terms: KOH onychomycosis, culture onychomycosis, nail biopsy, tinea unguium, tinea unguis, nail fungus, and onychomycosis combined with sensitivity, specificity, and predictive value. A total of 21 different search strategies were used.

The search was conducted in 5 multidisciplinary databases (Scopus, EBSCO, ScienceDirect, PubMed, and Lilacs) and an open Google search. Some of the search syntaxes used were (nail fungus [Title/Abstract]) AND specificity, onychomycosis and (predictive value), hongos de uñas AND sensibilidad, TI tinea unguium AND predictive value, TITLE-ABS-KEY ((onychomycosis AND (sensitivity OR specificity OR predictive value))). A search for articles found in the references of the recovered texts was also conducted, and consultation with experts took place to include unpublished research data.

#### Screening

The inclusion criteria were that the search terms were included in the title, abstract and/or keywords, the original journal articles were in English, Portuguese, or Spanish, and the research had been conducted in humans. The main criterion was the evaluation of diagnostic tests for onychomycosis, with accurate data on sensitivity and specificity, or predictive values for one or more of the three tests evaluated to estimate all parameters of a thorough diagnostic evaluation.

#### Selection

The exclusion criteria were studies describing overviews of onychomycosis, studies describing techniques and methods, and studies on clinical and/or therapeutic characterisations.

#### Inclusion

The resulting articles were subjected to an analysis of the full text, and the necessary information was extracted for the meta-analysis. At this stage, a qualitative and quantitative synthesis was performed as indicated in the analysis section [17].

### Reproducibility analysis and methodological quality assessment

The protocol was independently applied by two investigators to ensure the reproducibility of the search and study selection. At this stage, discrepancies were resolved by consensus. The extraction was also performed independently using an Excel file prepared with the study variables (author, year of publication, journal, country, age, gender, sensitivity, specificity, positive and negative predictive values, inclusion and exclusion criteria for study participants, true positives and negatives, and false positives and negatives) of each test evaluated. At this stage, reproducibility was ensured via calculation of kappa values for qualitative variables and the intraclass correlation coefficient for quantitative variables to obtain values of 1.00.

The quality of the studies was evaluated according to the criteria of the Quality Assessment of Diagnostic Accuracy Studies (QUADAS) tool. Potential biases included the selection of patients, the control and test, blinding in the interpretation and the applicability of the results according to the type of patients and tests studied [18, 19].

### Statistical analysis

For each of the three tests, validity was assessed through the parameters sensitivity, specificity, positive and negative likelihood ratios, Youden index (J), area under the ROC curve, performance of positive and negative predictive values, diagnostic odds ratio (OR), and accuracy or efficiency with the proportion of correctly diagnosed patients, all with their respective 95% confidence intervals. Satisfactory results for the diagnosis of onychomycosis were determined to be a negative likelihood ratio < 0.3, positive likelihood ratio > 4.0, area under the curve > 0.85, diagnostic OR much greater than 1.0 (for this study > 20), and predictive values and a proportion of correctly diagnosed patients > 70%.

A database with information extracted from each study was built and analysed with the meta-analysis of studies of evaluations of diagnostic and screening tests (Meta-DiSc) software with a significance level of 0.05. This software uses the Chi-square, DerSimonian-Laird (random effects model [REM]), Cochran-Q, Tau^2^, and inconsistency (I^2^) tests for the analysis of heterogeneity of sensitivity, specificity, likelihood ratios, diagnostic OR, and ROC curve, which are estimated as a combined measure using a random effects model. Additionally, the Epidat program was used to assess the predictive values, Youden index (J), test accuracy, and the prevalence of the disease with each test.

Finally, a meta-regression analysis was performed for the diagnostic assessment parameters based on the methodological quality of the studies. The studies were grouped into high-quality studies with scores of 13 and 14 in the QUADAS tool and moderate or average quality studies that complied 10 or less of the 14 criteria of this tool.

## Results

**Fig. 1:**
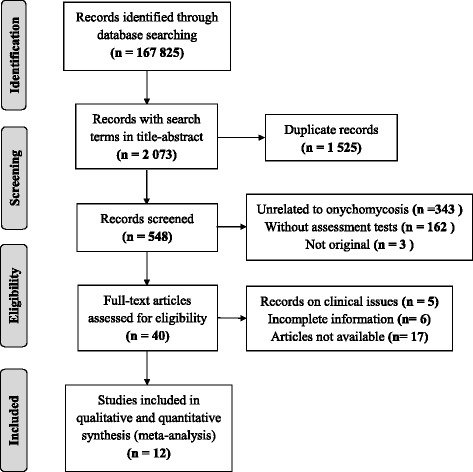
Study selection flow Diagram

Based on the search terms, 167,825 studies were found, of which only 2,073 included the terms in the title, abstract, or keywords. Of these, only 12 fulfilled the research protocol, and the others were excluded based on the protocol, as shown in Fig. 1. In addition to the 12 studies that fulfilled the search and selection protocol, a study conducted by the researchers was added, which corresponded to unpublished research.

Only 5 studies reported the place of study as follows: one in Iran [20], one in Germany [21], one in Taiwan [22], one in the United States [13], and one in Colombia [23]. All of the studies included in this meta-analysis defined the study population as subjects with clinical suspicion or clinical signs of onychomycosis, and the diagnostic positivity or reference criterion was considered to be any clinical suspicion of onychomycosis and at least one positive test for the condition.

The clinical criteria applied to patients in the included studies were not undergoing antifungal therapy 1 or 3 months prior to the study [13, 14, 24], not presenting dermatological diseases such as psoriasis, lichen planus, or other nail dystrophies [11, 14, 22, 24], and presenting a more serious clinical form than proximal subungual onychomycosis.

Regarding the clinical presentations, all studies reported some degree of dystrophy as an inclusion criterion but only 3 specified the number of patients broken down by type of lesion. For instance, in the study of Haghani et al. [20], the distal subungual form was more frequent (*n =* 88), followed by the white superficial (*n =* 5), proximal subungual (*n =* 5), proximal distal (*n =* 1), and total onychodystrophy (*n =* 1) forms. Karimzadegan-Nia et al. [14] reported more cases of total onychodystrophy (*n =* 77), followed by the distal subungual (*n =* 14), and white superficial (*n =* 5) forms. Finally, Lawry et al. [11] also included a greater number of patients with total onychodystrophy (*n =* 59), followed by the white superficial (*n =* 3) and distal subungual (*n =* 1) forms.

Table 1 describes some characteristics of the included studies, which were published between 2000 and 2016. A total of 2,858 subjects were studied. In the studies that reported the distribution of patients by gender, a greater proportion of men (57.1%) was found, the age range was wide (1–98 years), and the most common aetiological agents were dermatophytes (53.3% in all studies that reported the frequency of the fungi identified), followed by non-dermatophyte moulds (28.2%), and yeasts (18.5%).

**Table 1:** Study description

Author	Year	N	Gender		Age range in years (Mean)	Agents		
			#W^a^	#M^b^		#D^c^	#Y^d^	#NDM^e^
Lawry et al. [11]	2000	63	29	34	33–93	13	1	7
Borkowski et al. [13]	2001	50	31	19	11–98 (56)	NR	NR	NR
Weinberg et al. [26]	2003	105	NR	NR	NR	NR	NR	NR
Karimzadegan-Nia et al. [14]	2007	96	54	42	NR	9	13	3
Hsiao et al. [22]	2007	88	41	47	18–80 (50^±16^)	NR	NR	NR
Shenoy et al. [28]	2008	101	61	40	16–80 (45)	11	2	22
Alkhayat et al. [25]	2009	141	NR	NR	NR	28	15	30
Wilsmann-Theis et al. [21]	2011	1146	367	779	(56)	NR	NR	NR
Haghani et al. [20]	2013	101	79	22	NR	5	23	32
Jung et al. [27]	2015	493	222	271	(55^±16^)	130	1	19
Jeelani et al. [15]	2015	216	104	112	1–90 (36^±17^)	70	30	26
Hajar et al. [24]	2015	192	NR	NR	NR	29	6	NR
Velasquez et al. [23]	2016	66	50	16	19–87 (55^±16^)	13	16	24
		2858	1038	1382	0	308	107	163

Absolute frequency of ^a^Women ^b^Men ^c^Dermatophytes ^d^Yeasts ^e^Non-dermatophyte moulds

**Fig. 2:**
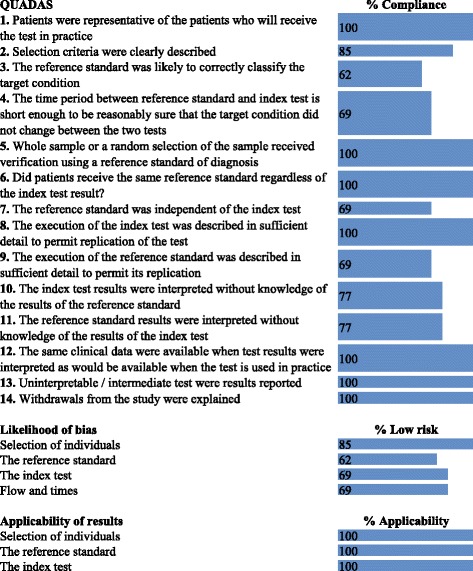
Assessment of the quality and risk of bias of the included studies

Regarding the assessment of the methodological quality, five studies met the 14 criteria of the QUADAS tool [20, 21, 23–25], four met 13 criteria [13–15, 26], and the other studies met 10 [27], 9 [11], and 8 criteria [22, 28]. The least applied criteria that were most related to potential bias were those related to the correct classification of the standard and to the independence between the application and interpretation of the evaluated test and the standard without affecting the applicability of the results to the clinical scenarios in which they were traditionally used (Fig. 2).

**Fig. 3:**
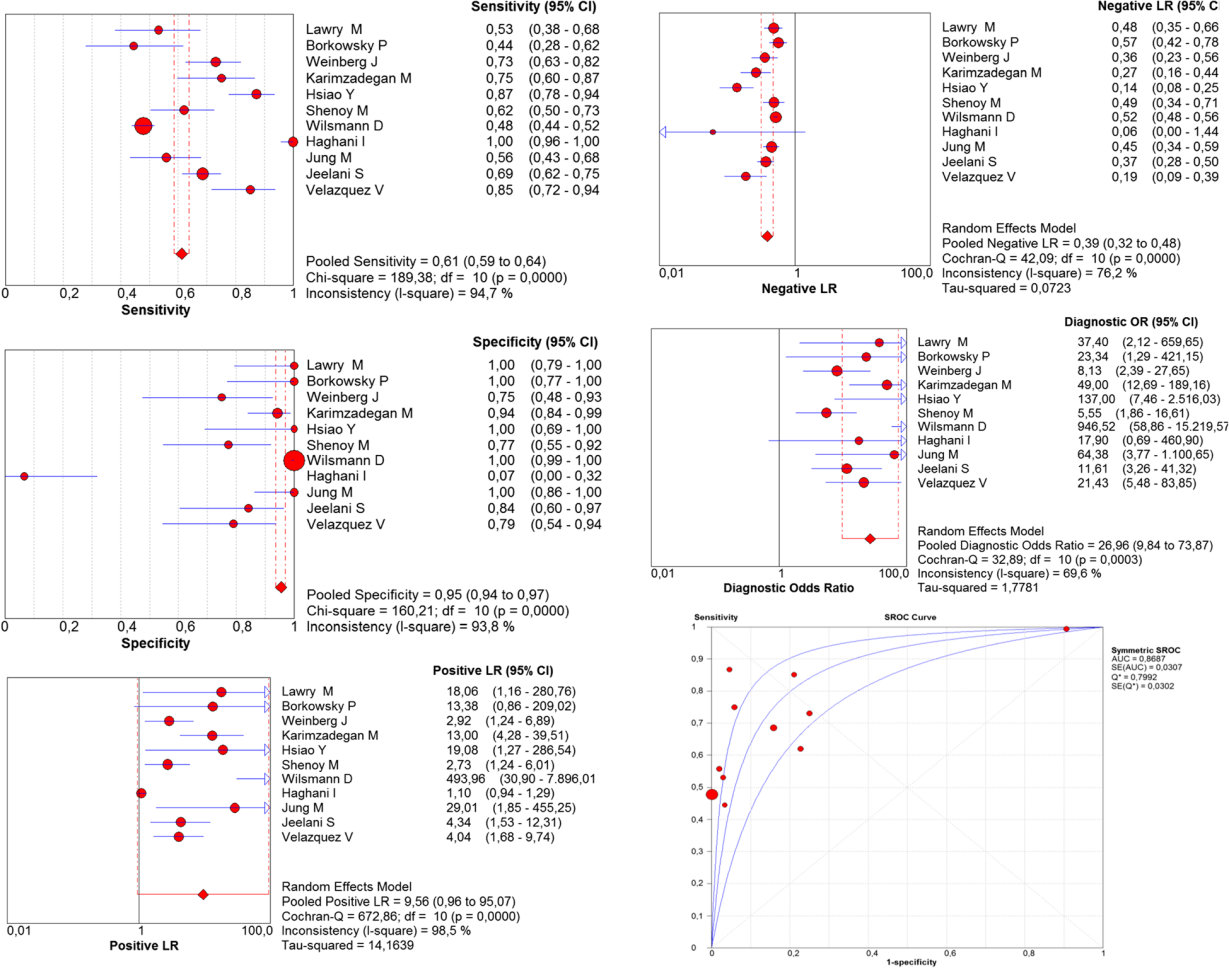
Meta-analysis of direct KOH examination for the diagnosis of onychomycosis (Sensitivity, Specificity, Positive Likelihood Ratios, Negative Likelihood Ratios, Diagnostic odds ratio, ROC curve)

In the meta-analysis of the direct KOH examination, heterogeneity was found in all of the evaluated parameters (p Chi-square, and Cochran-Q <0.05). In the combined measure under the random effects model, the sensitivity was 61% (95% CI = 59–64; I^2^ = 94.7%), specificity was 95% (95% CI = 94–97; I^2^ = 93.8%), positive likelihood ratio was 9.6 (95% CI = 1–95; I^2^ = 98.5%), negative likelihood ratio was 0.4 (95% CI = 0.3–0.5; I^2^ = 76.2%), diagnostic OR was 27 (95% CI = 10–74; I^2^ = 69.6%), area under the curve was 0.87 (Fig. 3), positive predictive value was 96.3% (95% CI = 96.2–96.4), negative predictive value was 55.9% (95% CI = 55.8–56.0), and accuracy was 72.8 (95% CI = 72.7–72.9) (Table 2).

**Table 2:** Comparison of the diagnostic evaluation parameters for direct KOH examination, culture, and biopsy according to the methodological quality of the studies

	KOH (95% CI)	Culture (95% CI)	Biopsy (95% CI)
Positive subjects: True Positives/False Negatives			
Total	857/545	966/745	1,483/288
High quality	677/453	684/448	975/236
Average quality	180/92	282/297	508/52
Negative subjects: True Negatives/False Positives			
Total	690/33	808/6	881/105
High quality	622/28	643/5	709/92
Average quality	68/5	165/1	172/13
Sensitivity			
Total	61 (59–64)	56 (54–59)	84 (82–86)
High quality	60 (57–63)	60 (58–63)	81 (78–83)
Average quality	66 (60–72)	49 (45–53)	91 (88–93)
Specificity			
Total	95 (94–97)	99 (98–100)	89 (87–91)
High quality	96 (94–97)	99 (98–100)	89 (86–91)
Average quality	93 (85–98)	99 (97–100)	93 (88–96)
Positive likelihood ratio			
Total	9.6 (1.0–95.1)	17.3 (6.1–48.8)	7.2 (2.8–18.9)
High quality	8.5 (0.3–255.7)	17.7 (3.4–91.8)	4.4 (1.4–13.8)
Average quality	9.6 (1.8–51.9)	16.6 (5.4–50.8)	24.3 (0.9–631.9)
Negative likelihood ratio			
Total	0.4 (0.3–0.5)	0.45 (0.4–0.5)	0.2 (0.1–0.3)
High quality	0.4 (0.3–0.5)	0.4 (0.3–0.5)	0.3 (0.2–0.4)
Average quality	0.4 (0.2–0.6)	0.5 (0.4–0.7)	0.1 (0.0–0.21)
Diagnostic OR			
Total	27.0 (9.8–73.9)	44.7 (17.9–111.5)	44.6 (13.0–153.5)
High quality	28.0 (7.4–106.3)	53.9 (12.7–229.1)	16.4 (4.2–64.8)
Average quality	25.5 (4.4–159.7)	32.3 (10.0–104.3)	249.1 (75.5–822)
Positive predictive value			
Total	96.3 (96.2–96.4)	99.4 (99.3–99.5)	93.4 (93.3–93.5)
High quality	96.0 (95.9–96.1)	99.3 (99.2–99.4)	91.4 (91.3–91.5)
Average quality	97.3 (97.0–97.6)	99.6 (99.5–99.8)	97.5 (97.4–97.6)
Negative predictive value			
Total	55.9 (55.8–56.0)	52.0 (51.9–52.1)	75.4 (75.3–75.5)
High quality	57.9 (57.8–57.9)	58.9 (58.8–59.0)	75.0 (74.9–75.1)
Average quality	42.5 (42.1–42.9)	35.7 (35.6–35.8)	76.8 (76.5–77.0)
Youden index (J)			
Total	0.57	0.56	0.73
High quality	0.56	0.60	0.69
Average quality	0.59	0.48	0.84
Efficiency			
Total	72.8 (72.7–72.9)	70.3 (70.2–70.4)	85.75 (85.7–85.8)
High quality	73.0 (72.9–73.1)	74.55 (74.5–74.6)	83.7 (83.6–83.8)
Average quality	71.9 (71.7–72.1)	60.0 (59.9–60.1)	91.3 (91.2–91.4)
Prevalence% (95% CI)			
Total	66.0 (65.9–66.1)	67.8 (67.7–67.9)	64.2 (64.2–64.3)
High quality	63.5 (63.4–63.6)	63.6 (63.5–63.7)	60.2 (60.1–60.3)
Average quality	78.8 (78.7–79.0)	77.7 (77.6–77.8)	75.2 (75.1–75.3)

**Fig. 4:**
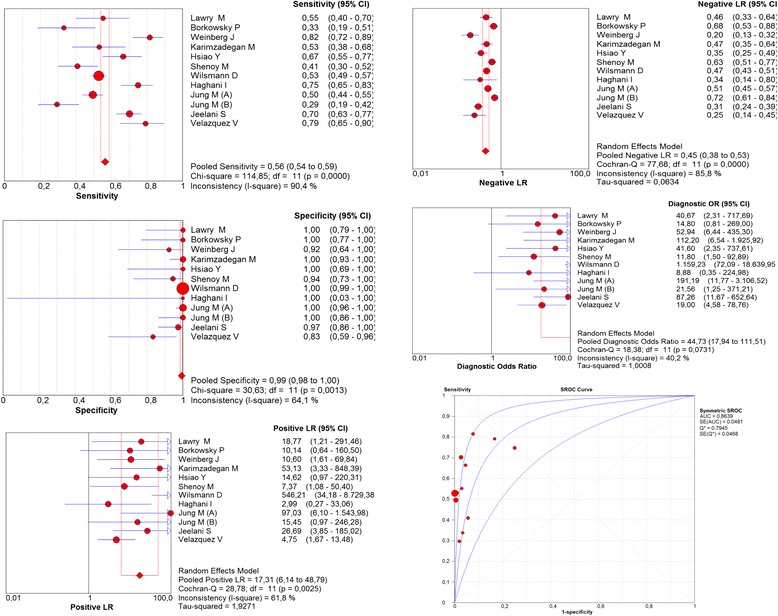
Meta-analysis of culture for the diagnosis of onychomycosis (Sensitivity, Specificity, Positive Likelihood Ratios, Negative Likelihood Ratios, Diagnostic odds ratio, ROC curve)

For culture, the overall sensitivity was 56% (95% CI = 54–59; I^2^ = 90.4%), specificity was 99% (95% CI = 98–100; I^2^ = 64.1%), positive likelihood ratio was 17.3 (95% CI = 6–49; I^2^ = 61.8%), negative likelihood ratio was 0.4 (95% CI = 0.3–0.5; I^2^ = 85.8%), diagnostic OR was 45 (95% CI = 18–111; I^2^ = 40.2%), area under the curve was 0.86 (Fig. 4), positive predictive value was 99.4% (95% CI = 99.3–99.5), negative predictive value was 52.0% (95% CI = 51.9–52.1), and accuracy was 70.3 (95% CI = 70.2–70.4) (Table 2).

**Fig. 5:**
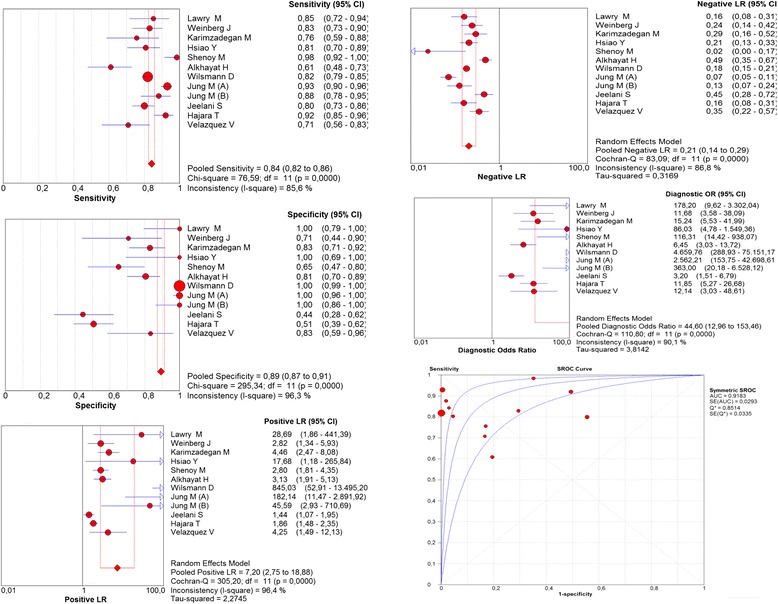
Meta-analysis of biopsy for the diagnosis of onychomycosis (Sensitivity, Specificity, Positive Likelihood Ratios, Negative Likelihood Ratios, Diagnostic odds ratio, ROC curve)

For biopsy, the overall sensitivity was 84% (95% CI = 82–86; I^2^ = 85.6%), specificity was 89% (95% CI = 87–91; I^2^ = 96.3%), positive likelihood ratio was 7.2 (95% CI = 3–19; I^2^ = 96.4%), negative likelihood ratio was 0.2 (95% CI = 0.1–0.3; I^2^ = 86.8%), diagnostic OR was 45 (95% CI = 13–153; I^2^ = 90.1%), area under the curve was 0.92 (Fig. 5), positive predictive value was 93.4% (95% CI = 93.3–93.5), negative predictive value was 75.4% (95% CI = 75.3–75.5), and accuracy was 85.75 (95% CI = 85.7–85.8) (Table 2).

Table 2 shows the overall diagnostic evaluation parameters, the parameters broken down by the methodological quality of the studies, and the number of individuals analysed in the different subgroups. Generally, the quality of the articles was not related to the conclusion on the validity and performance of the three diagnostic tests, with the exception of the sensitivity, negative predictive value, and positive predictive value for biopsy. In the moderate quality studies, the following differences were found compared to the total group: higher sensitivity for direct KOH examination and biopsy and lower sensitivity for culture, lower positive predictive value for KOH and culture, and higher positive and negative predictive values for biopsy. Finally, the efficiency or accuracy of the three tests was influenced by the methodological quality of the studies, with a lower value in the moderate quality studies for KOH and culture and a higher value for biopsy.

## Discussion

This meta-analysis presents a comprehensive evaluation of the validity, performance, and accuracy of diagnostics tests based on the evaluation of 2,858 individuals with clinical suspicion of onychomycosis, of which 2,125 were analysed with direct KOH examination, 2,525 with culture, and 2,757 with biopsy. The evaluation was performed on individuals over a wide age range, with a similar gender distribution, different causative agents, and from different countries in Europe, Asia, and the Americas. Many of the advantages of meta-analyses were available, such as analysing a broader reference population, increasing the statistical power of the analyses, improving the external validity or extrapolation of the results, and producing scientific evidence of greater validity, reproducibility, and cost-effectiveness compared to individual studies [29].

Meta-analyses of diagnostic tests help determine their validity, performance, and accuracy, limit or evaluate bias, minimise random findings, and generate conclusions of greater scientific quality [30]. For onychomycosis, reviews are available on risk factors and comorbidities [31], treatments [32], and disease overview [33]. However, no similar studies have evaluated the three traditional diagnostic methods (culture, biopsy, and direct KOH examination), which demonstrates the importance of this study.

Nail clipping with PAS staining (biopsy) was the most sensitive test, with an overall sensitivity of 84%; the highest sensitivity was obtained by Shenoy et al. [28] with 98% and the lowest by Alkhayat et al. with 61% [25]. Overall, all studies reported a sensitivity > 60% for nail clipping with PAS staining. This finding is contrary to the result observed for culture, which had an overall sensitivity of 56%, with the lowest sensitivity of 29% reported by Jung et al. [27] and the highest of 82% reported by Weinberg et al. [26]. Nail clipping with PAS staining is useful to confirm the presence of fungi in the nail plate and its invasion by visualisation of fungal structures [23]. Although culture was found to be the least sensitive test in this study, this test should not be ignored in the diagnosis of onychomycosis because it is the only one of the three tests that can detect the aetiological agent with greater accuracy and provides highly useful information to decide on the most appropriate antifungal therapy.

In contrast to the sensitivity reported in the studies, in which culture was the least valid test, the present study found that culture was the most specific test, with an overall result of 99% and several reports of 100% [13, 14, 20–22, 27]. These high specificity values may be biased because some studies reported 100% for this criterion as well as for the positive predictive value. These studies included the test evaluated in the standard, indicating that false positive results would never be obtained for the evaluated test.

Regarding sensitivity and specificity, it should be taken into account that these aspects are *a priori* intrinsic properties or probabilities of the tests that are not fully useful when applied in a clinical setting (i.e., they do not provide the certainty with which a clinician could determine whether a positive or negative result comes from an infected or healthy person) [34]. This information can be obtained using predictive values, which are the most useful clinical parameters [35]. Specifically in this study, all three tests showed excellent performance in infected individuals, with positive predictive values > 90%; however, the same finding was not observed for the negative predictive values, because only biopsy had an acceptable negative predictive value. This finding could be explained by the influence of the prevalence of the screened event on these parameters, which was very high in the case of onychomycosis [2].

Sensitivity and specificity are intrinsic test characteristics whose individual interpretations may differ when one result is high and the other is not, and predictive values are influenced by the prevalence of the screened infection or disease. Although other parameters are available for the diagnostic evaluation to overcome these limitations, they are rarely used in the evaluation of tests for onychomycosis. These parameters are the likelihood ratio, test accuracy, diagnostic OR, and Youden index (J). Likelihood ratios combine sensitivity and specificity to indicate the degree of certainty with which an infected patient can be detected in the presence of infection or a healthy individual in its absence [36]. Accuracy reflects the likelihood of valid results in all healthy and sick individuals tested. Diagnostic OR correlates test results with the presence of the disease. The Youden index (J) combines sensitivity and specificity and provides a global measure of validity and test agreement or disagreement [37].

Overall, all of the tests showed low utility when analysed separately. Nail clipping with PAS staining showed the highest likelihood ratios. With a positive likelihood ratio (PLR) of 7.20 and negative likelihood ratio (NLR) of 0.21; the best PLR was reported by Haghani et al. [20] with 845.03 and the best NLR by Jung et al. [27] with 0.07. Similar results were shown for the OR with 44.6, second only to the culture OR with 44.7, allowing the conclusion that these tests are the most useful to discriminate between healthy and infected individuals with no significant differences between them. Similarly, the area under the curve (AUC) for this test was 0.92, suggesting that it was the most valid test; similar results were obtained for the Youden index (J) and efficiency, confirming that nail clipping with PAS staining is the test with the greatest utility of the three tests evaluated.

In the meta-regression analysis, few evaluation parameters were influenced by the methodological quality of the studies. However, in this review, it was not possible to perform subgroup analyses based on other relevant variables, such as the degree or type of dystrophy, the causative agent, and the progression time of the lesion, among other microbiological, epidemiological, and demographic characteristics; this issue is one of the main limitations of this study.

## Conclusion

The diagnostic tests independently evaluated in this meta-analysis show acceptable validity, performance, and efficiency, with nail clipping with PAS staining outperforming the other two tests. However, the results indicate the need to combine the three tests to establish a diagnosis given their complementarity in terms of being able to identify the affected patients, causative agent, and degree of invasiveness.

## Abbreviations

AUC: Area under the curve
HIV: Human immunodeficiency virus
I^2^: Inconsistency
J: Youden index
KOH: Potassium hydroxide
MALDI-TOF: Matrix - assisted laser desorption/ionization time-of-flight
Meta-DiSc: Meta-analysis of studies of evaluations of diagnostic and screening tests
NLR: Negative likelihood ratio
OR: Odds ratio
PAS: Periodic acid-schiff
PCR: Polymerase chain reaction
PLR: Positive likelihood ratio
PRISMA: Preferred reporting items for systematic reviews and meta-analyses
QUADAS: Quality assessment of diagnostic accuracy studies
REM: Random effects model
ROC: Receiver operating characteristic

## References

[1] Mendoza N, Palacios C, Cardona N, Gómez L (2012). Onicomicosis: afección común de difícil tratamiento. Rev Asoc Colomb Dermatol.

[2] Relloso S, Arechavala A, Guelfand L, Maldonado I, Walker L, Agorio I (2012). Onychomycosis: multicentre epidemiological, clinical and mycological study. Rev Iberoam Micol.

[3] Thomas J, Jacobson GA, Narkowicz CK, Peterson GM, Burnet H, Sharpe C (2010). Toenail onychomycosis: an important global disease burden. J Clin Pharm Ther.

[4] Lone R, Bashir D, Ahmad S, Syed A, Khurshid S (2013). A study on clinico-mycological profile, aetiological agents and diagnosis of onychomycosis at a government medical college hospital in kashmir. J Clin Diagn Res.

[5] Balleste R, Mousques N, Gezuele E (2003). Onicomicosis. Revisión del tema. Rev Med Uruguay.

[6] Ameen M, Lear JT, Madan V, Mohd Mustapa MF, Richardson M (2014). British Association of Dermatologists’ guidelines for the management of onychomycosis 2014. Br J Dermatol.

[7] Cavallera E, Asbati M (2006). Onicomicosis por hongos filamentosos no dermatofitos. Dermatología Venezolana.

[8] López O, Torres J (1999). Especies fúngicas poco comunes responsables de onicomicosis. Rev Iberoam Micol.

[9] Gupta AK, Simpson FC (2013). Diagnosing onychomycosis. Clin Dermatol.

[10] Stewart CL, Rubin AI (2012). Update: nail unit dermatopathology. Dermatol Ther.

[11] Lawry MA, Haneke E, Strobeck K, Martin S, Zimmer B, Romano PS (2000). Methods for diagnosing onychomycosis: a comparative study and review of the literature. Arch Dermatol.

[12] Zanardi D, Holthausen D, Da Silva A, Quirino M, De Souza J (2008). Avaliação dos métodosdiagnósticos para onicomicose. An Bras Dermatol.

[13] Borkowski P, Williams M, Holewinski J, Bakotic B (2001). Onychomycosis: an analysis of 50 cases and a comparison of diagnostic techniques. J Am Podiatr Med Assoc.

[14] Karimzadegan-Nia M, Mir-Amin-Mohammadi A, Bouzari N, Firooz A (2007). Comparison of direct smear, culture and histology for the diagnosis of onychomycosis. Australas J Dermatol.

[15] Jeelani S, Ahmed QM, Lanker AM, Hassan I, Jeelani N, Fazili T (2015). Histopathological examination of nail clippings using PAS staining (HPE-PAS): gold standard in diagnosis of onychomycosis. Mycoses.

[16] Guevara M, Urcia F, Casquero J. Manual de procedimientos y técnicas de laboratorio para la identificación de los principales hongos oportunistas causantes de micosis humanas. Lima: Ministerio de Salud, Instituto Nacional de Salud. 2007.

[17] Moher D, Liberati A, Tetzlaff J, Altman DG, Prisma Group (2009). Preferred reporting items for systematic reviews and meta-analyses: the PRISMA statement. Ann Intern Med.

[18] Abraira V (2006). Sesgos en los estudios sobre pruebas diagnósticas. SEMERGEN-Medicina de Familia.

[19] Whiting P, Rutjes AW, Reitsma JB, Bossuyt PM, Kleijnen J (2003). The development of QUADAS: a tool for the quality assessment of studies of diagnostic accuracy included in systematic reviews. BMC Med Res Methodol.

[20] Haghani I, Shokohi T, Hajheidari Z, Khalilian A, Aghili SR (2013). Comparison of diagnostic methods in the evaluation of onychomycosis. Mycopathologia.

[21] Wilsmann-Theis D, Sareika F, Bieber T, Schmid-Wendtner MH, Wenzel J (2011). New reasons for histopathological nail-clipping examination in the diagnosis of onychomycosis. J Eur Acad Dermatol Venereol.

[22] Hsiao YP, Lin HS, Wu TW, Shih HC, Wei SJ, Wang YL (2007). A comparative study of KOH test, PAS staining and fungal culture in diagnosis of onychomycosis in Taiwan. J Dermatol Sci.

[23] Velasquez V, De Bedout C, Cardona J, Cano L (2016). Evaluaciòn de la biopsia ungueal como herramienta de apoyo en el diagnóstico de onicomicosis en un laboratorio de referencia de la ciudad de Medellin, Colombia.

[24] Hajar T, Fernandez-Martinez R, Moreno-Coutino G, Vasquez Del Mercado E, Arenas R (2016). Modified PAS stain: a new diagnostic method for onychomycosis. Rev Iberoam Micol.

[25] Alkhayat H, Al-Sulaili N, O’Brien E, McCuaig C, Watters K (2009). The PAS stain for routine diagnosis of onychomycosis. Bahrain Medical Bulletin.

[26] Weinberg JM, Koestenblatt EK, Tutrone WD, Tishler HR, Najarian L (2003). Comparison of diagnostic methods in the evaluation of onychomycosis. J Am Acad Dermatol.

[27] Jung MY, Shim JH, Lee JH, Lee JH, Yang JM, Lee DY (2015). Comparison of diagnostic methods for onychomycosis, and proposal of a diagnostic algorithm. Clin Exp Dermatol.

[28] Shenoy MM, Teerthanath S, Karnaker VK, Girisha BS, Krishna Prasad MS, Pinto J (2008). Comparison of potassium hydroxide mount and mycological culture with histopathologic examination using periodic acid-Schiff staining of the nail clippings in the diagnosis of onychomycosis. Indian J Dermatol Venereol Leprol.

[29] Manterola C, Carlos; Vial M, Pineda V, Sanhueza A. Revisión sistemática de la literatura con diferentes tipos de diseños. International Journal of Morphology. 2009;27:1179–86

[30] González J, Balaguer A. Revisión sistemática y metaanálisis (I): conceptos básicos. Evidencias en Pediatría. 2007;3(4):1–10.

[31] Elewski BE, Tosti A (2015). Risk factors and comorbidities for onychomycosis: implications for treatment with topical therapy. J Clin Aesthet Dermatol.

[32] Gupta AK, Daigle D, Foley KA (2015). Network meta-analysis of onychomycosis treatments. Skin Appendage Disord.

[33] Vlahovic TC (2016). Onychomycosis: evaluation, treatment options, managing recurrence, and patient outcomes. Clin Podiatr Med Surg.

[34] Escrig-Sos J, Martinez-Ramos D, Miralles-Tena JM (2006). Diagnostic tests: basic concepts for their correct interpretation and use. Cir Esp.

[35] Cerda LJ, Cifuentes AL (2010). Clinical use of diagnostic tests (Part 1): analysis of the properties of a diagnostic test. Rev Chilena Infectol.

[36] Cifuentes L, Cerda J (2010). Clinical use of diagnostic tests (Part 2): clinical application and usefulness of a diagnostic test. Rev Chilena Infectol.

[37] Szklo M, Nieto F (2003). Epidemiología intermedia: conceptos y aplicaciones.

